# Different adaptive patterns of wheat with different drought tolerance under drought stresses and rehydration revealed by integrated metabolomic and transcriptomic analysis

**DOI:** 10.3389/fpls.2022.1008624

**Published:** 2022-10-13

**Authors:** Liangjie Lv, Xiyong Chen, Hui Li, Jinan Huang, Yuping Liu, Aiju Zhao

**Affiliations:** Crop Genetics and Breeding Laboratory of Hebei, Institute of Cereal and Oil Crops, Hebei Academy of Agriculture and Forestry Sciences, Shijiazhuang, China

**Keywords:** wheat, drought, rehydration, metabolome, transcriptome

## Abstract

Wheat as a staple food crop is enduring ever-frequent intermittent and changing drought with the climate change. It is of great significance to highlight the adaptive approaches under such variable conditions at multiple levels to provide a comprehensive understanding of drought tolerance and facilitate the genetic breeding of wheat. Therefore, three wheat lines with different drought tolerance (drought-tolerant mutant *Mu* > common wheat CK > drought susceptible mutant *mu*) were analyzed under moderate and severe drought stresses as well as rehydration. Samples were subjected to transcriptomic and metabolomic profiling in combination with physiological and biochemical determination. The moderate drought stress rendered 198 and 115 differentially expressed metabolites (DEMs) in CK and *Mu*, respectively. The severe drought stress rendered 166, 151 and 137 DEMs in CK, *Mu* and *mu*, respectively. The rehydration rendered 150 and 127 DEMs in CK and *Mu*. 12,557 and 10,402 differentially expressed genes (DEGs) were identified for CK and *Mu* under moderate drought stress, respectively. 9,893, 7,924, and 9,387 DEGs were identified for CK, *Mu*, and *mu* under severe drought stress, respectively. 13,874 and 14,839 were identified in CK and Mu under rehydration, respectively. Metabolomics results showed that amino acid was the most differentially expressed metabolites, followed by phenolic acids. Flavonoids played an important role in drought tolerance. Most enriched pathways under drought included biosynthesis of secondary metabolites, metabolic pathways and photosynthesis. Metabolites and genes involved in osmotic regulation, antioxidase activities, and ABA signaling were more enriched in *Mu* than in CK and mu. Various drought-responsive genes and metabolites in *Mu* showed different trends with those in CK and *mu*. Increased amino acids biosynthetic capability and ROS scavenging ability resulted from higher antioxidase activities and increased flavonoids may be the mechanisms underlying the drought tolerance characteristic of *Mu*. Recovery from reversible ROS damage and rapid amino acid biosynthesis may contribute to the rapid recovery of *Mu*. The present study provides new insights for mechanisms of wheat under complex drought conditions.

## Introduction

Drought is one of the most important environmental factors and severely impact crop growth, yield and quality (Krasensky and Jonak, 2012; Zhang et al., 2014; Tatar et al., 2020). After long terms of evolution, plants have developed various strategies to cope with deleterious effects of drought, which deserves in-depth investigation. The main coping strategies to drought employed by plants can be summarized as drought escape, drought avoidance, and drought tolerance (Du et al., 2018; Osmolovskaya et al., 2018; Li et al., 2021). The escape strategy generally involves a seasonal response, including life or growth cycle adjustment (Basu et al., 2016). In the drought avoidance strategy, plants enhance water uptake and reduce water loss. Drought tolerance is mediated by osmotic adjustment (such as accumulation of osmotin and protective proteins), extension of antioxidant capacity (including enzymatic and non-enzymatic activities), and development of desiccation tolerance (such as cell wall hardening; Zhang, 2007).

Wheat is one of the staple crops in the world and endures frequent drought stresses. Drought tolerance strategies have also been documented in wheat. Like other plants, wheat under drought synthesizes and accumulates more osmotic regulatory substances, such as proline, betaine, and other inorganic ions (Francki and Appels, 2002). Genetically modified engineering confirmed that genes encoding delta1-pyrroline-5-carboxylate synthetase (P5CS; Vendruscolo et al., 2007) and 1-phosphate mannitol dehydrogenase (mlt D; Abebe et al., 2003) could result in drought tolerance improvement of wheat. Protective proteins also play a role in the drought tolerance process of wheat. Sivamani et al. (2000) introduced *HVA1*, the gene encoding barley group 3 LEA protein, into wheat, and found that under drought conditions, the biomass and water use efficiency of transgenic wheat were increased, and the survival rate was improved. Moreover, wheat transcription factors such as *DREB2* (Morran et al., 2011), *WRKY2* (Gao et al., 2018), and *NAC69* (Xue et al., 2011) are also reported to be related to drought tolerance of wheat. The expression of *TaFER-5B* was induced by drought. Overexpression of this gene improved the drought resistance, heat resistance, oxidation resistance and high iron resistance of wheat plants, which was mainly due to the improved ability to scavenge reactive oxygen species, increased catalase (CAT) and glutathione reductase (GR) activities, and decreased H_2_O_2_ content (Zang et al., 2017).

Plants are often grown in a changing environment with alternating dry and wet conditions, especially for crops with artificial interference (Yang et al., 2022), such as wheat. In other words, intermittent drought occurs more frequently in actual production. The rapid recovery ability of plants after rehydration, on the one hand, can quickly reduce the damage caused by drought stress, and at the same time reduce the impact of drought on plant yield with different degrees of compensation effect, which is of great significance to plant production. However, reports addressing responses to progressive drought and recovery upon rehydration are relatively limited (Abid et al., 2018).

OMICS techniques including metabolomics, transcriptomics, proteomics, and ionomics, provide a wider scope of mechanisms underlying the biological process compared with conventional methods (Budak et al., 2015). They contribute a lot to the identification and characterization of genes, proteins, metabolites, and ions involved in signaling pathways of interest. With the progress in methodology and the decrease in cost, these techniques have been widely adopted in drought-related researches (Wilkins et al., 2010; You et al., 2019; Xu et al., 2021). Great progress in investigations into wheat under drought stresses have also been made (Xiao et al., 2012; Reddy et al., 2014; Zhan et al., 2014). Proteomics and metabolomics of leaf tissues from spring-wheat showed that photosynthetic proteins and enzymes involved in sugar and nitrogen metabolism, as well as capacity of detoxifying harmful molecules were of significance during drought response of wheat. Ma et al. (2019) adopted transcriptome to investigate the differentially expressed genes and enriched pathways during drought-sensitive period under field conditions in bread wheat, and pointed out that genes encoding tubulins, 6-phosphogluconate dehydrogenase (PGD6), cuticular wax-associated proteins, and heat shock proteins were greatly involved. Li et al. (2022) revealed a novel regulation of drought resistance during germination in wheat by integrated metabolome and transcriptome. They showed that the DEGs that participated in the mTOR and alpha-linolenic acid metabolism pathways were considered candidate DEGs related to drought resistance.

Overall, we contend that drought stress studies should be extended to explore the differences of cultivars/lines with as many approaches as possible, and drought intensities as well as rehydration should be taken into consideration to gain more insights of wheat under complex drought situations. Therefore, the present study was carried out to characterize the physiological, biochemical, metabolomic and transcriptomic responses of three wheat lines with different drought tolerance to different intensities of drought as well as rehydration. Our study may provide a comprehensive understanding of the underlying mechanisms of wheat response to drought and rehydration, and facilitate the breeding of drought-tolerant cultivars.

## Materials and methods

### Plant materials and treatments

The common wheat cultivar Jimai 418, designated as CK, and its two ethyl methane sulfonate (EMS)-induced mutants, namely drought-tolerant mutant *Mu* and drought-susceptible mutant *mu*, were taken as experimental materials. The details of the EMS mutagenesis can be found in our previous study (Lv et al., 2020). The experiment was carried out in an incubation room of the Institute of Grain and Oil Crops, Hebei Academy of Agriculture and Forestry Sciences (114.38°, 38.23° E N, 50 m). All wheat plants were grown in a greenhouse under 23°C/14 h light and 15°C/10 h dark. Seeds of the three lines were surface-sterilized with 0.2% HgCl_2_ for 20 min, followed by rinsing thoroughly with distilled water, and dried on filter papers. The seeds were planted in free-draining plastic pots filled with clay loam soil. Three pots were prepared for each line with different treatments. The moisture was kept at 70–80% field capacity by irrigating with tap water until the drought stress treatments. Thirty-five days after the planting (tillering stage), wheat seedlings of CK and *Mu* were subjected to normal irrigation (about 70–80% field capacity), moderate drought (about 50–60% field capacity) and severe drought (about 30–40% field capacity) for 10 days, respectively (seedlings undergone the treatments were designated as CK1, CK2, CK3, *Mu*1, *Mu*2, and *Mu*3). Wheat seedlings of *mu* were subjected to normal irrigation and severe drought stress (designated as *mu*1 and *mu*3). Rehydration was carried out for CK3 and *Mu*3 to allow a 3-day recovery (seedlings undergone the rehydration were designated as CK4 and *Mu*4, respectively). At the end of each treatment, fresh leaves were collected for physiological and biochemical analysis, or immediately frozen in liquid nitrogen and stored at –80°C for further metabolomic and transcriptomic analysis.

### Determination of soluble sugar content, soluble protein content and enzymic antioxidant activities

Fresh leaves (0.5 g) were homogenized at 4°C in 100 mM potassium phosphate buffer (pH 7.0) supplemented with 1 mM EDTA and 1% polyvinylpyrrolidone (*w*/*v*). The homogenates were centrifuged at 13,000 × *g* for 10 min. The soluble sugar content was determined according to Wood (Wood, 2002). The soluble sugar content was determined by the Coomassie brilliant blue G250 method (Pierce and Suelter, 1977). The catalase (CAT) activity was measured by the hydrogen peroxide reduction method and the peroxidase (POD) activity was measured by the guaiacol method (Chance and Maehly, 1955); the superoxide dismutase (SOD) activity was measured by nitrous blue Tetrazole photoreduction method (Giannopolitis and Ries, 1977); malondialdehyde (MDA) was determined using a detection kit for MDA (Nanjing Jiancheng Bioengineering institute, Nanjing, China) according to the manufacturer’s instructions.

### RNA extraction and sequencing

TRIzol reagent (Invitrogen, CA, United States) was used to extract total RNA of the leaves. The RNA was quantified with a Qubit fluorometer (Invertrogen) and its integrity was verified by an Agilent 2100 Bioanalyzer (Agilent, CA, United States) and polyacrylamide gel electrophoresis. Sequencing libraries were generated using NEBNext^®^Ultra^™^ RNA Library Prep Kit for Illumina^®^ (NEB, United States) following the manufacturer’s instructions. The libraries were quality-controlled, pooled, and subjected to sequencing on an Illumina Hiseq Xten platform (Illumina, United States). Sequences containing adaptors, and those with more than 10% unknown nucleotides and 50% low-quality sequences (*Q* value ≤ 20) were removed from the data sets to obtain clean reads. These clean reads were then mapped to the wheat reference genome of “Chinese spring”^1^ by HISAT2. Mapped reads and transcript length were normalized and gene expression were estimated by fragments per kilobase of transcript per million fragments mapped (FPKM). Differential expressed genes (DEGs) of two groups were identified with the DESeq2 R package (Love et al., 2014; Varet et al., 2016) using the standard of false discovery rate (FDR) < 0.01 and fold change (FC) > 2 or < 0.5. DEGs were then annotated based on the KO (KEGG Orthologue database),^2^ and Gene Ontology (GO).^3^ We used KOBAS (Xie et al., 2011) software to test the statistical enrichment of differential expression genes in KEGG pathways. The R packages pheatmap version 1.0.12 and VennDiagram version 1.7 were used to generate the heatmaps and Venn diagrams.

### Identification and analysis of metabolites

The frozen leaves were grounded to powder and 100 mg powder was extracted overnight at 4°C with 0.6 ml 70% aqueous methanol, centrifuged at 10,000 g for 10 min, absorbed in CNWBOND Carbon-GCB SPE Cartridge (250 mg, 3 ml; ANPEL, Shanghai, China) and filtrated with a 0.22 μm SCAA-104 (ANPEL, Shanghai, China). The obtained extracts were then subjected to metabolites analysis with an ultra-performance liquid chromatography (UPLC)-ESI-MS/MS system (UPLC, ExionLC AD; MS, Applied Biosystems 6,500 Triple Quadrupole). The parameters were set as previously described (Zhu et al., 2017), and a mixture of supernatant from each biological sample was used as a quality control (QC) sample to evaluate the stability of the system. The data was scaled and subjected to principal component analysis (PCA). Metabolites with variable important in projection (VIP) greater than or equal to 1 and absolute Log_2_FC (fold change) greater than or equal to 1 were deemed as significantly regulated and annotated against the KEGG database. *K*-means clustering was carried out with R software.

### Quantitative real-time PCR analysis

The RNA was transcribed into cDNA using the Reverse Transcription System (Promega, United States), and qRT-PCR was carried out with the SYBR Premix Ex Taq (TAKARA, Japan) on a LightCycler480 instrument (Rotkreuz, Switzerland). The expression was calculated using the 2^–∆∆Ct^ method (Livak and Schmittgen, 2001) and normalized to ACTIN. Primers used are summarized in Supplementary Table S1.

### Statistical analysis

The physiological and biochemical parameters, as well as the qRT-PCR data were analyzed using IBM SPSS Statistics version 23.0 (IBM, NY, United States). The data were presented as means ± standard error. Significant differences were identified using one-way ANOVA followed by the Duncan’s multiple range test (*p* < 0.05).

## Results

### Physiological and physiochemical changes during drought and recovery periods

**Figure 1:**
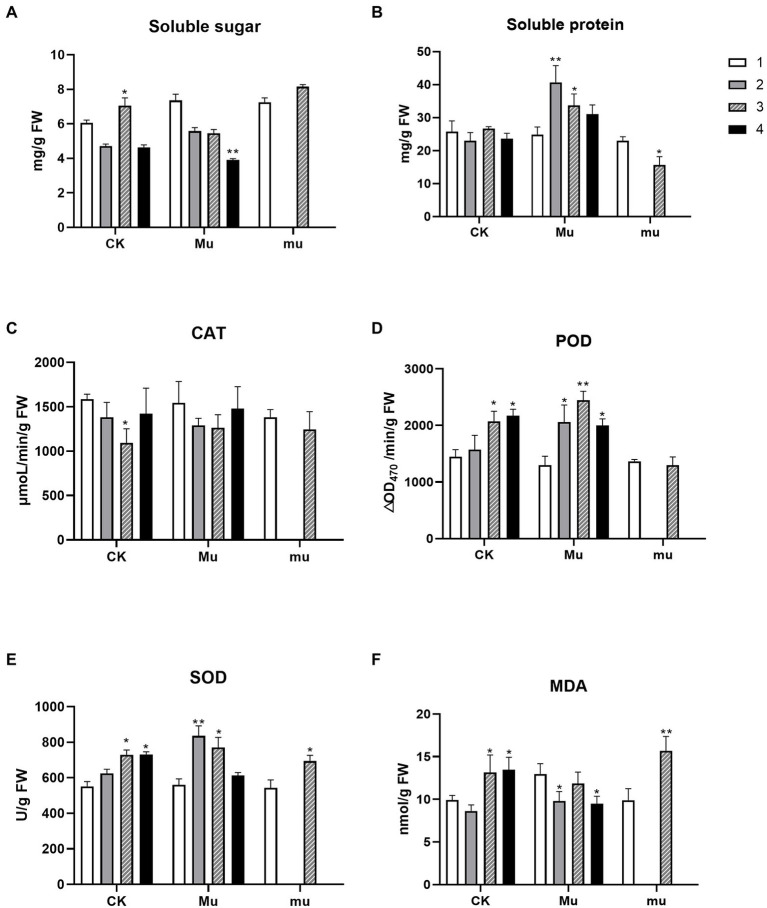
Physiological and biochemical changes of the three wheat lines during drought stresses and rehydration. Contents of soluble sugar **(A)**, soluble protein **(B)**, and MDA **(F)** and enzymic activities of CAT **(C)**, POD **(D)**, and SOD **(E)** of wheat under different treatments. The number 1–4 indicate different treatment. 1, pre-treatment; 2, moderate drought stress; 3, severe drought stress; 4, rehydration. Stars on the bar indicate significant difference as compared to the stage before drought. ^*^p < 0.05, ^**^p < 0.01.

Soluble sugar and protein are among the two important osmotic regulatory substances. A significant increase in soluble sugar only occurred in CK under severe drought stress, while the content of soluble sugar in *Mu* decreased both under drought stresses and rehydration, and the decrease reached significant (*p* < 0.01) under rehydration, as compared to *Mu*1 (Figure 1A). As for the soluble protein, *Mu* exhibited significant increase under drought stresses, and *mu* showed a significant decrease under severe drought (Figure 1B). During drought stresses, the activities of SOD and POD increased in all the wheat lines, except that the POD activity of *mu* remained unchanged (Figures 1C,D). No significant change was observed in the CAT activity of all wheat lines, except that the CAT activity in CK increased significantly under severe drought (Figure 1E). The MDA content increased significantly in both CK and *mu* under drought, but decreased significantly in *Mu* under moderate drought stress and rehydration (Figure 1F).

### Metabolic dynamics during drought stresses and recovery in wheat

**Figure 2:**
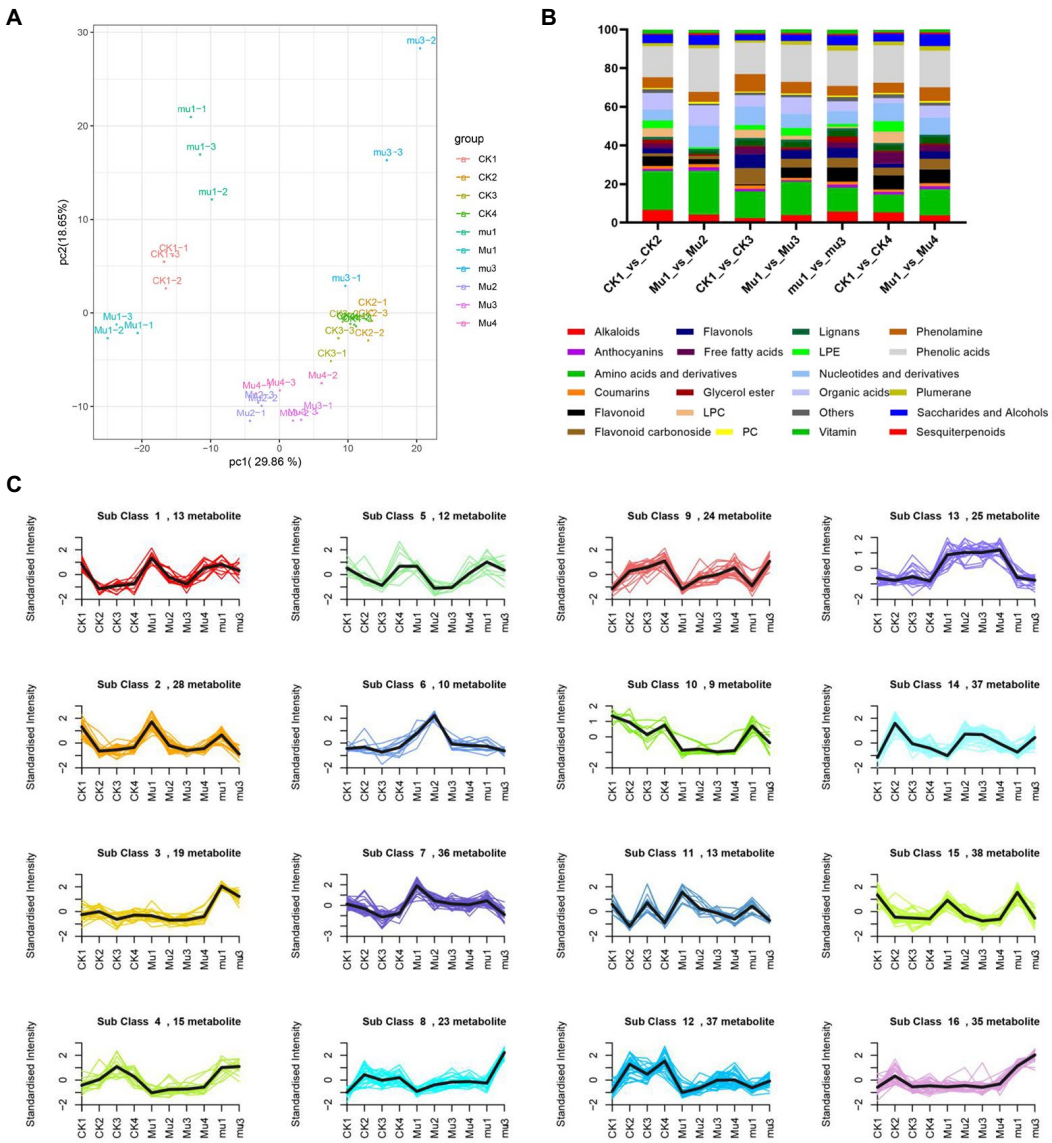
Metabolic dynamics during drought stresses and recovery in wheat. **(A)** PCA analysis of all samples. **(B)** Percentage of each kind of differentially expressed metabolites. **(C)** K-means clustering analysis of all the metabolites.

The metabolic dynamics of the three lines during moderate and severe drought stresses and rehydration were analyzed. The metabolites were detected by UPLC−ESI-MS/MS. A total of 553 metabolites for 30 samples were obtained, including 50 alkaloids, 74 amino acids and derivatives, 100 flavonoids, 21 lignans and coumarins, 77 lipids, 46 nucleotides and derivatives, 40 organic acids, 97 phenolic acids, and so on (Supplementary Table S2). To reveal the clear separation among the 10 groups of the three lines under different treatments, principal component analysis (PCA) was adopted. It was showed that the first principal component (PC1) explained 29.86% of the variation, which distinguished the untreated samples from samples under drought stresses and rehydration (Figure 2A). The second principle component (PC2) explained 18.65% of the variation, which separated *mu* with other samples. The biological replicates clustered together, suggesting a good correlation of replicates.

To identify key metabolites responding to drought stresses and rehydration, differentially expressed metabolites (DEMs) were screened under a standard of fold change ≥ 2 or ≤ 0.5 and VIP ≥ 1. The moderate drought stress rendered 198 and 115 DEMs in CK and *Mu*, respectively. The severe drought stress rendered 166, 151 and 137 DEMs in CK, *Mu* and *mu*, respectively. 150 and 127 DEMs were found in CK and *Mu* during rehydration, respectively, as compared to CK1 and *Mu*1 (Supplementary Table S3). The percentage of each kind of DEM is shown in Figure 2B. Under moderate drought stress, more amino acids and derivatives (22.6%) and phenolic acids (22.6%) were differentially accumulated in *Mu* than in CK (20.2 and 16.2%). During severe drought stress and rehydration, the situation was similar to that under moderate drought stress, that is, more amino acids and derivatives were differentially accumulated in *Mu* than in CK. Among the amino acids, L-proline plays an important role in drought tolerance. It increased in all the three lines during drought stresses, and recovered in CK but did not recover in *Mu* after rehydration (Supplementary Figure S1). Soluble sugars including sucrose, trehalose anhydrous, isomaltulose, and melibiose in *Mu* decreased during drought stresses and did not recover after rehydration, as compared with those in *Mu*1 (Supplementary Table S2).

Next, *K*-means clustering was performed to identify the metabolites that might contribute to the drought tolerance differences of the three lines. The metabolites were clustered into 16 sub classes. Most metabolites in the three lines showed similar responses to drought stresses and rehydration, but their fold changes were different. Among these sub classes, sub classes 6 and 13 were of particular interest, since metabolites in sub class 6 showed a dramatic increase under moderate drought stress and that in sub class 13 remained highly accumulated. Metabolites in the two sub classes included 14 phenolic acids and 9 flavonoids (Supplementary Table S4).

### Overall transcriptomic analysis of wheat during drought stresses and rehydration

**Figure 3:**
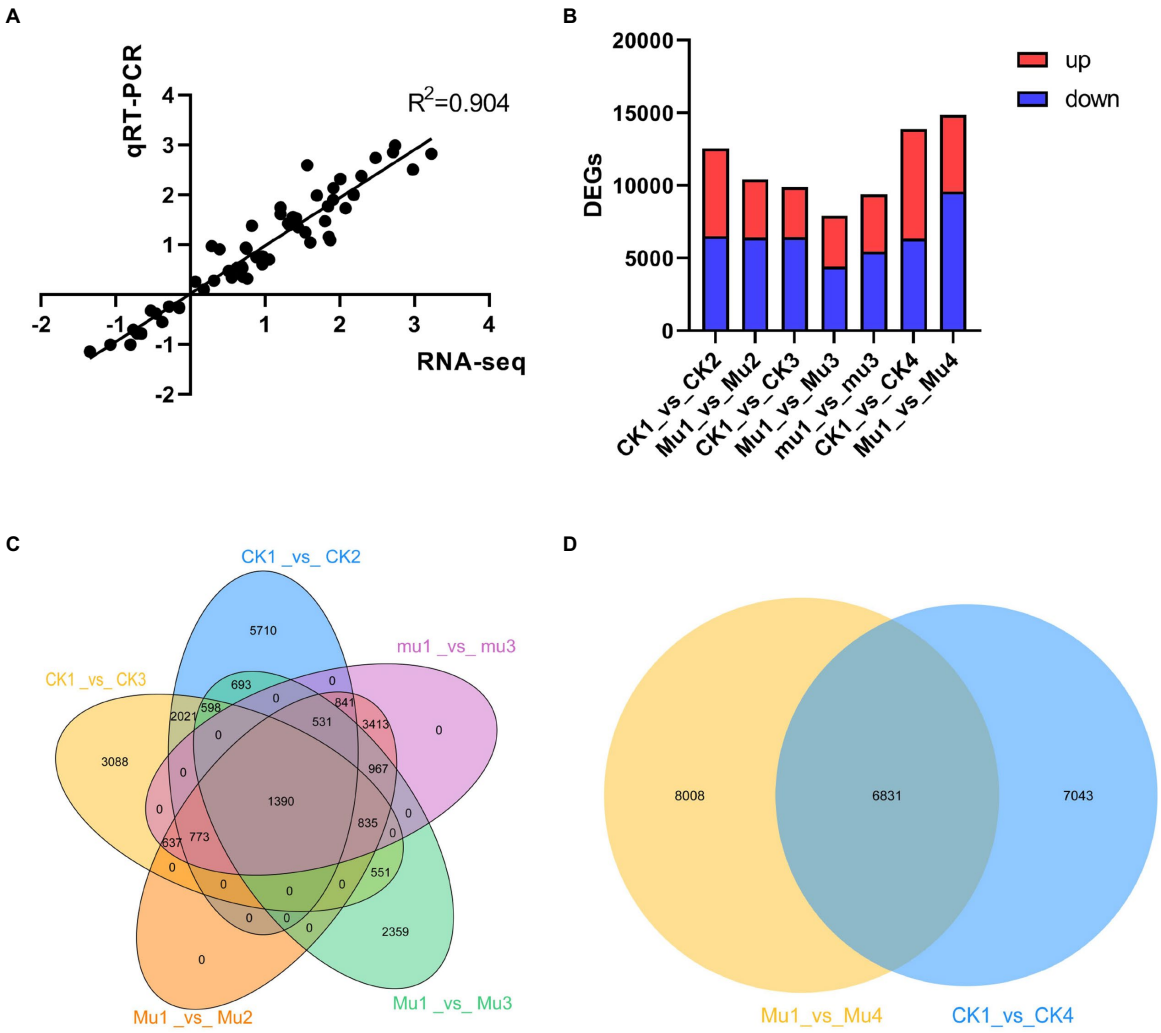
Overall transcriptomic analysis of wheat during drought stresses and recovery. **(A)** Correlation analysis of qRT-PCR and RNA seq results. **(B)** Statistics of DEGs during moderate and severe drought stresses and rehydration. **(C)** Venn diagram of DEGs during moderate and severe drought stresses. **(D)** Venn diagram of DEGs after rehydration.

To investigate how wheat lines with different drought tolerances cope with drought stresses and rehydration at transcriptomic levels, the samples were subjected to next-generation sequencing. A total of 2.73 billion clean reads were obtained for 30 samples, with over 11 Gb for each sample on average and the Q30 value reached as high as 93% (Supplementary Table S5). To validate the accuracy of the sequencing, quantitative real-time PCR (qRT-PCR) was carried out with 12 genes of interest. It was showed that the *R*^2^ was 0.904 (Figure 3A), suggesting the consistency of qRT-PCR and RNA-sequencing. Raw reads were uploaded to NCBI and the SRA accession number was PRJNA801431 and PRJNA800468. PCA was adopted to analyze the clear separation. The results were slightly different with that of metabolome, where samples under severe drought stress were separated with others, with the PC1 explaining 19.89% of the variation (Supplementary Figure S2). DEGs were then identified under the standard of FDR < 0.01 and FC > 2 or < 0.5. The moderate drought stress each rendered 12,557 and 10,402 DEGs in CK and *Mu*, compared with CK1 and *Mu*1, respectively. The severe drought stress each rendered 9,893, 7,924, and 9,387 DEGs in CK, *Mu*, and *mu*, as compared with CK1, *Mu*1 and *mu*1, respectively. There were 13,874 and 14,839 DEGs in CK1_vs_CK4, and *Mu*1_vs_*Mu*4 groups, respectively (Figure 3B). Thousand and three hundred and ninety DEGs were commonly expressed in all the three lines under drought stresses (Figure 3C), and 4,831 DEGs were identified to be common in CK1_vs_CK4, and *Mu*1_vs_*Mu*4 groups (Figure 3D).

### Differentially expressed genes involved in osmotic regulation

**Figure 4:**
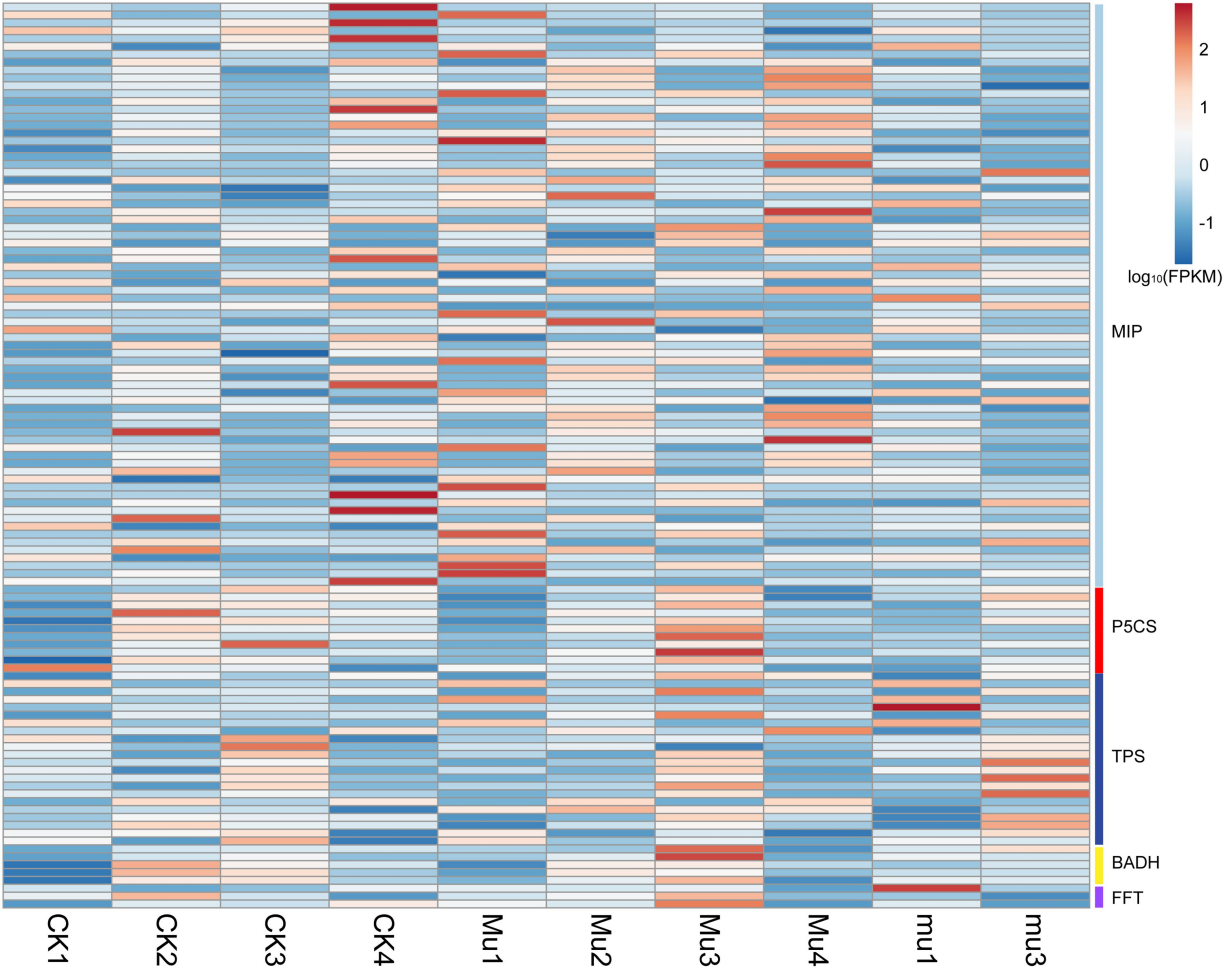
Heatmap showing the expression of DEGs related to osmotic regulation. Gene expression is normalized using FPKM and represents the means of three biological replicates. Expression values are presented as FPKM normalized log10-transformed counts. The color represents relative gene expression levels. While the horizontal direction shows the sample names, the vertical direction shows the names of genes involved in osmotic regulation. MIP: major intrinsic protein, P5CS: Delta-1-pyrroline-5-carboxylate synthase, TPS: trehalose-phosphate synthase, BADH: betaine aldehyde dehydrogenase, FFT: fructan 1-fructosyltransferase.

Osmotic regulation is an important approach adopted by plants during drought stresses and rehydration. To investigate how the osmotic regulation was carried out in different wheat lines at transcriptomic levels, DEGs involved in this process were identified (Figure 4). It was showed that genes encoding major intrinsic protein (MIP, also known as aquaporin), Delta-1-pyrroline-5-carboxylate synthase (P5CS), trehalose-phosphate synthase (TPS), betaine aldehyde dehydrogenase (BADH), and fructan 1-fructosyltransferase (FFT) were differentially expressed in all the three lines during drought stresses and rehydration, and it seemed that *Mu*3 had higher expression of the above genes than CK3 and *mu*3 (Figure 4; Supplementary Table S6). It is noted that genes encoding MIP in *Mu* had higher expression during rehydration than in other treatments.

### Differentially expressed genes involved in enzymic antioxidation

**Figure 5:**
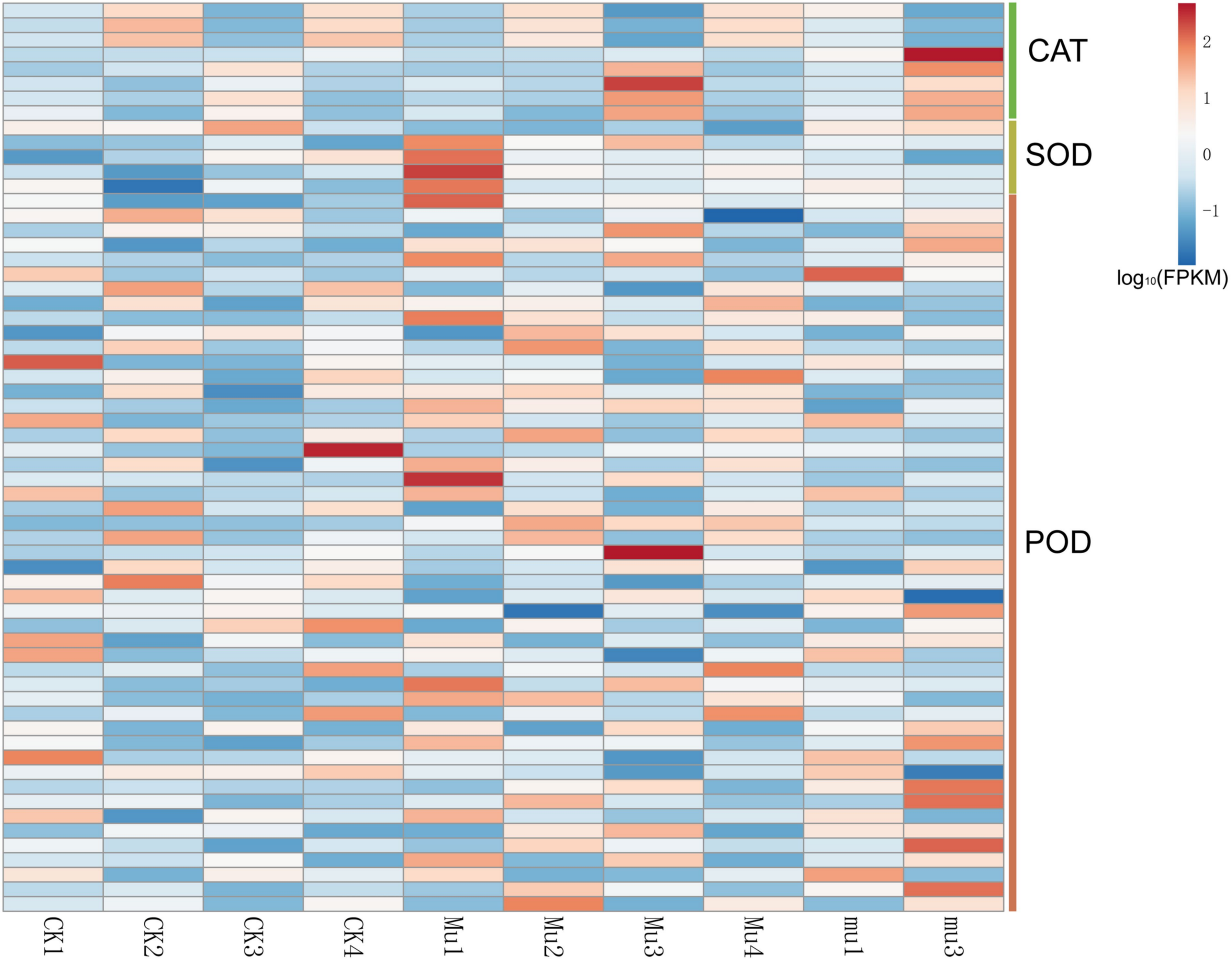
Heatmap showing the expression of DEGs related to enzymic antioxidant. Gene expression is normalized using FPKM and represents the means of three biological replicates. Expression values are presented as FPKM normalized log10-transformed counts. The color represents relative gene expression levels. While the horizontal direction shows the sample names, the vertical direction shows the names of genes involved in osmotic regulation. DEGs involved direct to those encoding catalase, superoxide dismutase, peroxidase, and glutathione peroxidase.

Genes relevant to antioxidase (FPKM > 1), including genes encoding CAT, POD, and SOD were found to be differentially expressed as expected (Figure 5 and Supplementary Table S7). Their overall expression was almost consistent with the activities of the enzymes they encoded. However, genes encoding different isozymes or subunits of the same antioxidase had different expression patterns. For example, although both *TraesCS6A02G041700* and *TraesCS6D02G048300* belonged to catalase, *TraesCS6A02G041700* was highly expressed in CK2, and *TraesCS6D02G048300* was highly expressed in CK3.

### Differentially expressed genes involved in flavonoids synthesis

**Figure 6:**
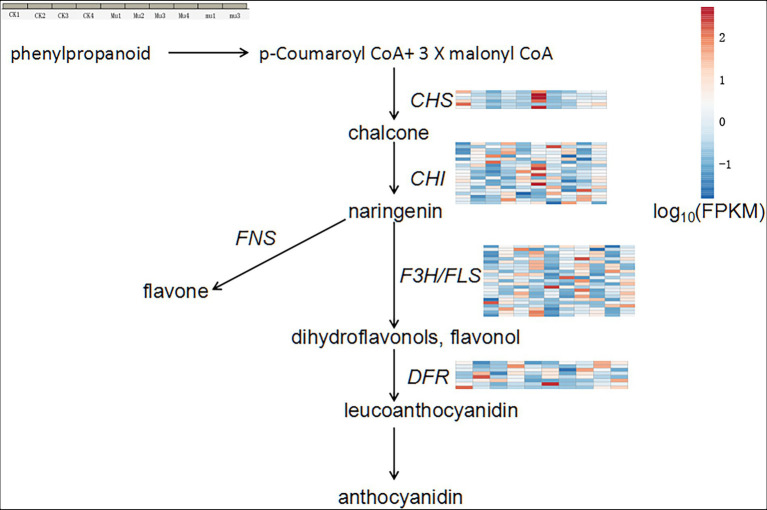
General anthocyanidin synthesis pathway and expression profile of DEGs involved in the pathway. Gene expression is normalized using FPKM and represents the means of three biological replicates. Expression values are presented as FPKM normalized log10-transformed counts. The color represents relative gene expression levels. While the horizontal direction shows the sample names, the vertical direction shows the names of genes involved in osmotic regulation. CHS, chalcone synthase; CHI, chalcone isomerase, F3H, flavanone 3-hydroxylase; FNS, flavone synthase; FLS, flavonol synthase; DFR, dihydroflavonol-4-reductase.

The metabolomics results showed that flavonoids were highly accumulated in *Mu*. To determine genes contributing to the accumulated flavonoids, we analyzed the expression profile of genes (FPKM > 1) involved in the general flavonoids biosynthesis pathway in plants (Figure 6 and Supplementary Table S8). It was showed that *Mu*2 and *Mu*3 had the highest expression in terms of DEGs encoding chalcone synthase (CHS), chalcone isomerase (CHI), flavanone 3-hydroxylase (F3H), flavonol synthase (FLS), dihydroflavonol-4-reductase (DFR). The trend was especially clear for CHS and CHI. Genes encoding flavone synthase (FNS) was not found to be differentially expressed.

### Differentially expressed genes involved in ABA signaling

**Figure 7:**
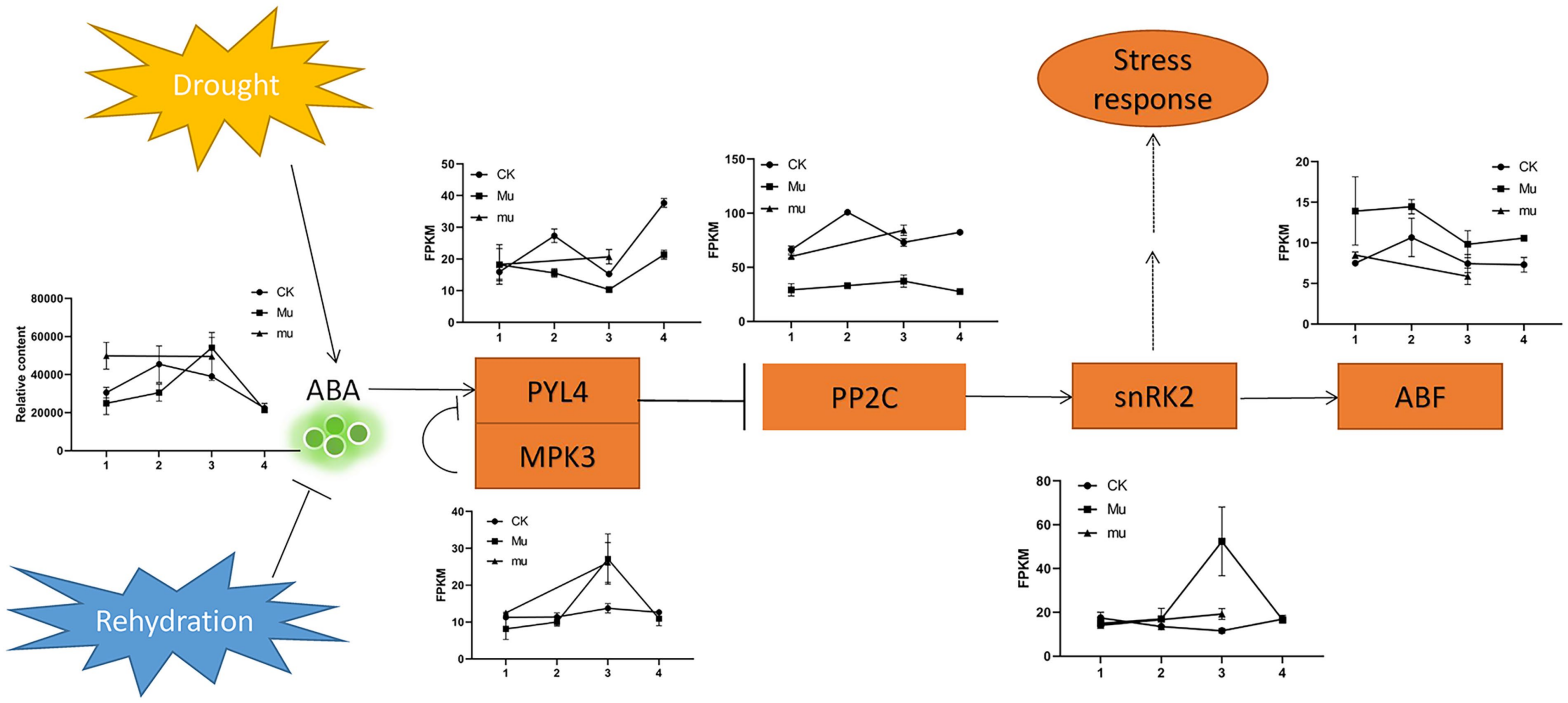
Expression pattern of DEGs involved in ABA signaling. PYL4, pyrabactin resistance 1-like 4; MPK3, mitogen protein kinase 3; PP2C, protein phosphatases 2C; snRK2, sucrose nonfermenting-1 (SNF1) related kinase 2; ABF, ABA-responsive element-binding factors. Rectangles indicate genes, Oval indicates biological process, arrows indicate promotion, bars (T) indicate inhibition, and dashed lines indicate postulated regulation.

Abscisic acid (ABA) is a sensitive indicator for drought and rehydration. It was up-regulated under drought in all the three lines, but the pattern was different. It increased with the drought degree in *Mu*, but increased then slightly decreased with the increasing drought degree in CK (Figure 7). Figure 7 shows a typical signaling pathway and representative expression pattern of genes encoding pyrabactin resistance 1-like 4 (PYL4), mitogen protein kinase 3 (MPK3), protein phosphatases 2C (PP2C), sucrose nonfermenting-1 (SNF1) related kinase 2 s (snRK2), and ABA-responsive element-binding factors (ABF). In *Mu*, the gene encoding PYL4 showed opposite trend with that encoding MPK3, which was consistent with the negative relationship of the two genes. The expression trends of the genes in *Mu* under different degrees of drought were quite different from that in CK and *mu*, which highlighted its drought tolerance.

### Differentially expressed genes involved in photosynthesis

**Figure 8:**
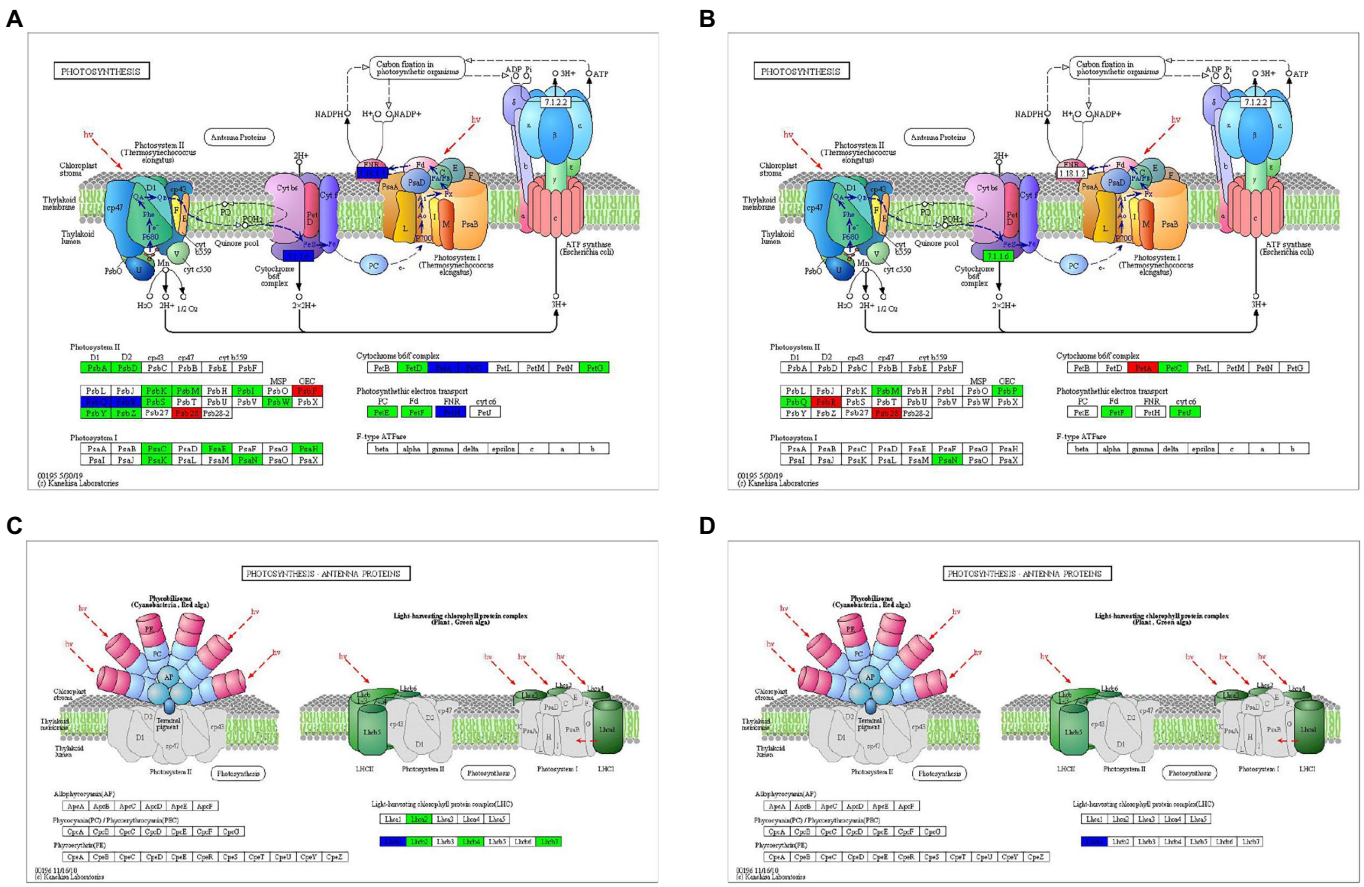
DEGs involved in photosynthesis during rehydration. DEGs involved in the “photosynthesis” pathway after rehydration in CK **(A)** and *Mu* **(B)**. DEGs involved in the “photosynthesis-antenna proteins” pathway after rehydration in CK **(C)** and *Mu* **(D)**.

A lot of DEGs participating PSI, PSII, cytochrome b6/f complex, photosynthetic electron transport, F-type ATPase, and light harvesting chlorophyll II protein complex were identified (Supplementary Figure S3, only *Mu*3_vs_*Mu*1 was showed as representatives, since the situation was similar in other groups). After rehydration, it was found that less photosynthetic genes were significantly down-regulated in *Mu* than in CK (Figure 8). The expression of genes encoding photosynthesis-antenna protein was almost totally recovered from drought after rehydration.

### Kyoto encyclopedia of genes and genomes and GO enrichment of DEGs during drought stresses and rehydration

**Figure 9:**
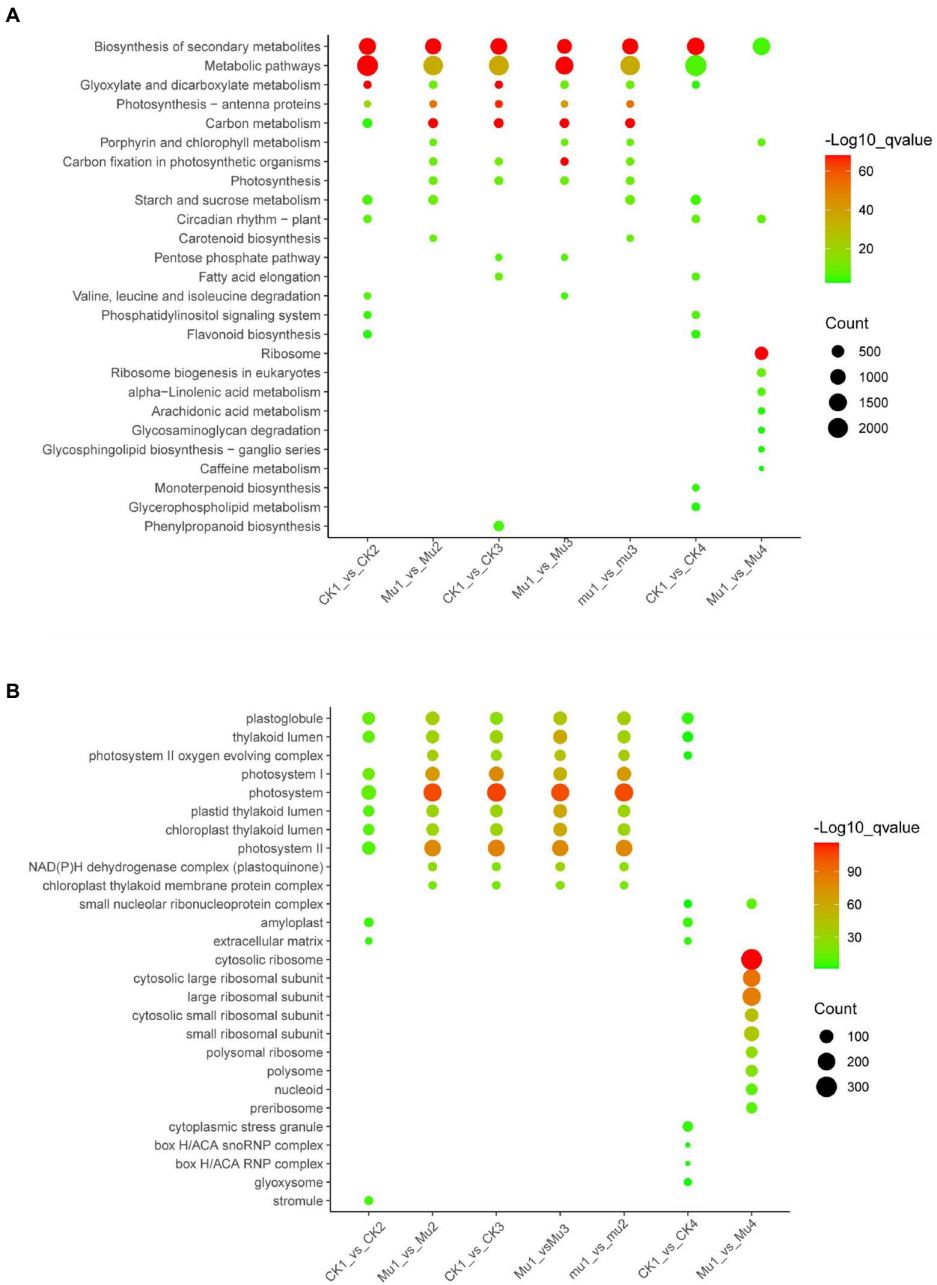
KEGG and GO enrichment of DEGs during drought stresses and rehydration. The most enriched top10 pathways and GO terms are displayed. **(A)** KEGG enrichment of DEGs during drought stresses and rehydration. **(B)** GO enrichment of DEGs during drought stresses and rehydration.

Kyoto encyclopedia of genes (KEGG) and genomes and GO enrichment of the DEGs was performed to gain more insights into the expression profile of the three lines during drought stresses and rehydration. The KEGG enrichment showed that biosynthesis of secondary metabolites (ko01110), metabolic pathway (ko01100), glyoxylate and dicarboxylate metabolism (ko00630), and carbon metabolism (ko01200) were among the common and most enriched pathways among all the three lines during drought stresses. The most enriched pathways in CK during rehydration were biosynthesis of secondary metabolites (ko01110), metabolic pathway (ko01100), glyoxylate and dicarboxylate metabolism (ko00630), starch and sucrose metabolism (ko00500), etc. The most enriched pathways in *Mu* during rehydration were ribosome (ko03010), biosynthesis of secondary metabolites (ko01110), carotenoid biosynthesis (ko00906), etc. (Figure 9A).

The GO enrichment results showed that GO terms involved in photosynthesis, including photosystem II oxygen evolving complex (GO: 0009654), photosystem I (GO: 0009522), photosystem (GO: 0009521), thylakoid lumen (GO: 0031977) and photosystem II (GO: 0009523), were among the most enriched in the three lines during drought stresses, with the enrich factors varied among different mutants. GO enrichment patterns were quite different between CK and *Mu* during rehydration. Amyloplast (GO: 0009501), cytoplasmic stress granule (GO: 0010494), and extracellular matrix (GO: 0031012) were among the most enriched GO terms in CK, while ribosomal related terms, such as cytosolic ribosome (GO: 0022626), cytosolic large ribosomal unit (GO: 0022625) and large ribsomal unit (GO: 0015934) were among the most enriched GO terms in *Mu* (Figure 9B).

### Correlation analysis of metabolome and transcriptome

**Figure 10:**
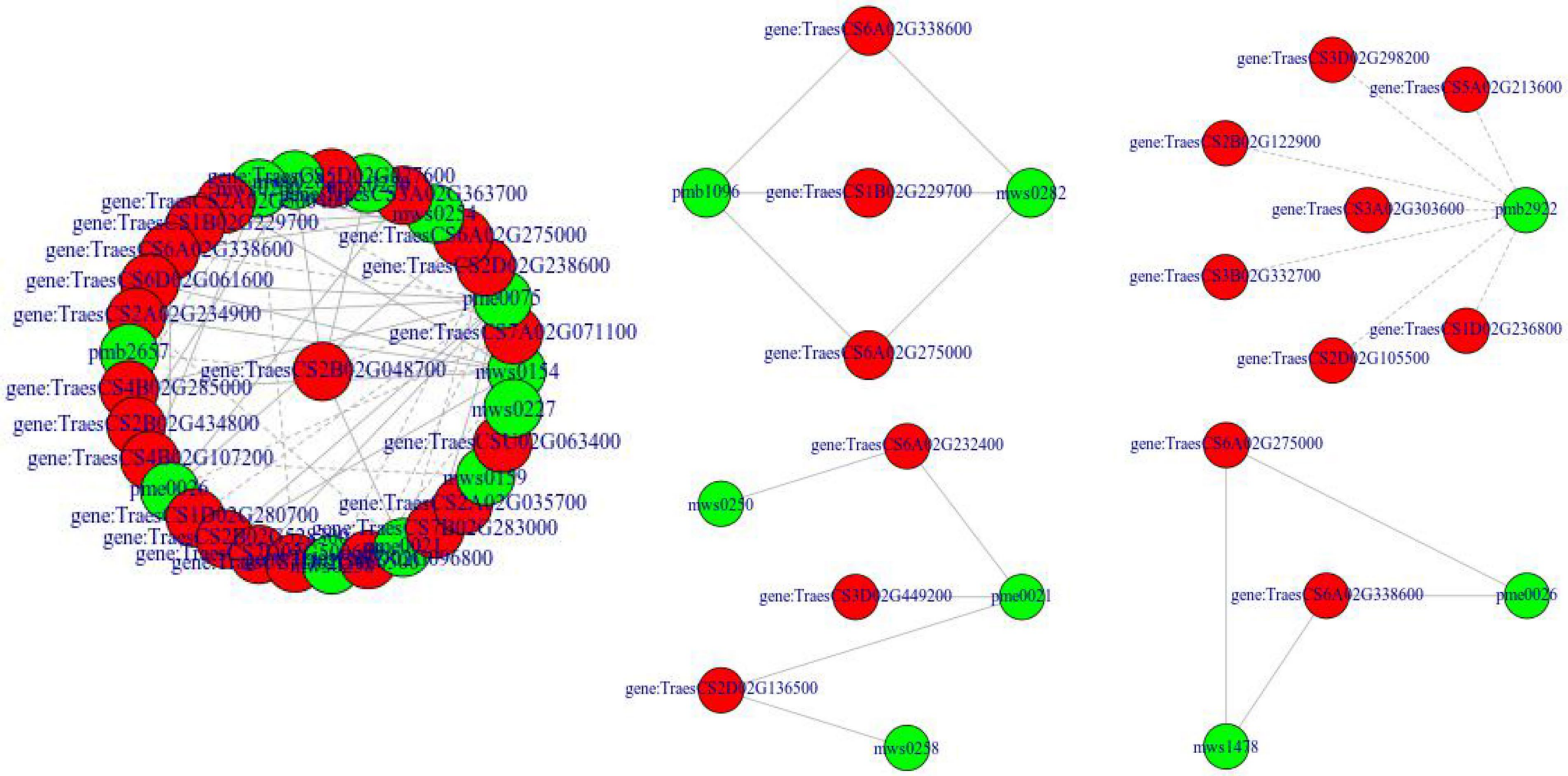
Network constructed based on the co-regulated pathways of metabolome and transcriptome. Red indicates genes, green indicates metabolites, solid line indicates positive relationship, and dash line indicates negative relationship.

To screen key pathways and genes involved in the response to drought and rehydration, we performed correlation analysis of metabolome and transcriptome. The co-regulated pathways are summarized in Table 1. No significant co-regulated pathway was found in CK1_vs_CK3, CK4_vs_*Mu*4, and *Mu*1_vs_*Mu*4. It was showed that Biosynthesis of secondary metabolites (ko01110), Biosynthesis of amino acids (ko01230), Cyanoamino acid metabolism (ko00460), Phenylalanine, tyrosine and tryptophan biosynthesis (ko00400), and Tropane, piperidine and pyridine alkaloid biosynthesis (ko00960) etc., were the significantly co-regulated pathways (*p* < 0.01). A network related to these pathways was constructed. The network revealed that various amino acids and their derivatives including shikimic acid (mws0154), L-phenylalanine (pme0021) and N-Acetyl-L-glutamic acid (pme0075) were positively correlated with genes encoding fructose-biphosphate aldolase (*TraesCS7B02G283000*), P5CS (*TraesCS3A02G363700*), and citrate synthase (*TraesCS5B02G416700*) (Figure 10).

**Table 1:** Co-regulated pathways according to the metabolome and transcriptome analysis.

Group	KEGG_pathway	ko_id	*p*-Value_gene	*p*-Value_meta	Gene count	Meta count
Ck1_vs_CK2	Biosynthesis of secondary metabolites	ko01110	0	0.049517903	1,309	53
	Glycerophospholipid metabolism	ko00564	9.96304E-05	0.007050157	105	10
	Cyanoamino acid metabolism	ko00460	0.000405749	0.029054387	90	6
	Phenylalanine, tyrosine and tryptophan biosynthesis	ko00400	0.001741824	0.002373248	37	9
	Tropane, piperidine and pyridine alkaloid biosynthesis	ko00960	0.004636233	0.012961524	28	7
	Ether lipid metabolism	ko00565	0.016238155	0.016858706	32	4
Mu1_vs_Mu2	Tryptophan metabolism	ko00380	0.012577873	0.005746905	79	8
	Starch and sucrose metabolism	ko00500	1.81577E-12	0.039731574	201	4
Mu1_vs_Mu3	Cyanoamino acid metabolism	ko00460	0.045165556	0.043430103	54	5
	Tropane, piperidine and pyridine alkaloid biosynthesis	ko00960	0.018237689	0.017365249	19	6
	Biosynthesis of secondary metabolites	ko01110	0	0.011639658	875	45
	Biosynthesis of amino acids	ko01230	0.012701418	0.008826043	113	18
mu1_vs_mu3	Tryptophan metabolism	ko00380	0.012577873	0.018888298	79	6

## Discussion

Although various strategies for wheat to cope with drought stress and rehydration have been characterized (Osmolovskaya et al., 2018), these strategies vary and are even contradict sometimes. It is believed that these variations may be due to the differences in cultivars, depth of investigation and strength of drought stresses, etc. Taking into consideration of the fact that wheat is enduring ever-frequent intermittent drought, we for the first time took advantage of a commonly planted wheat cultivar and its drought-tolerant mutant and drought susceptible mutant to study their physiological, biochemical, metabolomic and transcriptomic responses to different intensities of drought and rehydration after severe drought stress. The present study provides a comprehensive understanding of plant under complicated drought conditions and is expected to facilitate the genetic breeding of wheat with excellent drought tolerance.

Generally, plants having poor drought tolerance are more prone to be disturbed by drought due to absence of homeostasis mechanisms to mitigate the impact of water deficit (You et al., 2019). The overall results of transcriptome and metabolome in the present study showed that there were less genes and metabolites to be changing in *Mu* than in CK and *mu*, suggesting the disturbance existed at both transcriptional and metabolic levels. The hyper-responsive to abiotic stress at the molecular level in susceptible genotype was also reported in other plant species (Ma et al., 2016; Muthusamy et al., 2016; Svoboda et al., 2016).

Osmotic adjustment is a critical process for maintaining water status and physiological activity of plant cells (Krasensky and Jonak, 2012; Gurrieri et al., 2020). Osmotically active substances including soluble sugars, amino acids (especially proline), soluble protein, betaine, and organic acid have been well documented to be changing under drought stress (Paudel et al., 2016; Tschaplinski et al., 2019). These substances bring about stabilization of membranes, proteins, and other subcellular structures under osmotic stress. In the present study, the contents of proline in all the three lines increased under both moderate and severe drought stresses (Supplementary Figure S1), which is consistent with Abid et al. (2018) and Cao et al. (2021). In accordance with the metabolomic results, the gene encoding P5CS, a key enzyme for proline, was up-regulated in all the three lines during drought stresses. Interestingly, *mu* accumulated more proline during severe drought stresses than the others, and the proline contents in *mu* and CK decreased after rehydration but sustained high in *Mu*, which is not in agreement with previous reports where drought-tolerant cultivars had higher proline content (Abid et al., 2018). This indicates that although proline is an indicator for drought stress but is not a biomarker for drought tolerance, which is in contrast to the conclusion of Dien et al. (2019). We suggest that the linear regulation of proline may be a more reliable indicator when evaluating drought tolerance. Similarly, soluble sugar and protein respond to drought rapidly, but their dynamic changes vary among cultivars according to previous studies (Dien et al., 2019; Du et al., 2020; Islam et al., 2020). In the present study, total soluble sugar and oligoses were significantly decreased under drought stresses and did not recover after rehydration (Figure 1; Supplementary Figure S2). This may be due to the consumption of energy for coping with stresses and the weakened photosynthesis.

Water deficiency damages the balance between reactive oxygen species (ROS) generation and scavenging, eliciting oxidative stress, and up-regulation of ROS production (Cruz De Carvalho, 2008). Plants have developed a series of precise regulatory strategies to cope with the toxic effect of ROS during their long-period evolution, mainly involving enzymic and non-enzymic detoxification systems (Asensi-Fabado and Munné-Bosch, 2010). In the present study, SOD and POD activity in both CK and *Mu* were significantly increased under drought stresses and recovered to pre-treatment levels, which is in accordance with a previous study (Abid et al., 2018). However, unlike previous reported researches (Farooq et al., 2021), it seemed that CAT failed to play a role in coping with ROS, since only the CAT activity in CK3 significantly decreased (Figure 1). It was reported that exogenous application of some phenolic acid would result in decrease in CAT activity, while other phenolic acids increased antioxidase activities including CAT and SOD (Bhardwaj et al., 2017). Since some phenolic acids were significantly accumulated in *Mu* during drought stress (Figure 1C), we speculate that the decreased CAT activity was specifically inhibited by the highly accumulated phenolic acids. Among the non-enzymic substances, flavonoids have been recognized to have excellent ROS scavenging competent and respond to biotic and abiotic stress situations in *Scutellaria baicalensis Georgi* (Yuan et al., 2012), rice (Ithal and Reddy, 2004) and maize (Christie et al., 1994). It was found in the present study that flavonoids and phenolic acids were highly accumulated and responded quickly to drought stress (Figure 2C), so were the flavonoids synthetic genes, such as *CHS*, *CHI*, *DFR* and *FLS* (Figure 6). The results are in agreement with those of Ma et al. (2014) and Liu et al. (2013). Although there were more kinds of flavonoids and derivatives thereof differentially accumulated in *mu* (Figure 2B), they seemed to make little contribution to ROS scavenging. Anthocyanins such as petunidin-3-O-glucoside, cyanidin-3-O-(6″-p-Coumaroylglucoside), and petunidin-3-(6″-p-Coumaroylglucoside), and flavonoids such as apigenin-3-O-rhamnoside, and Chrysoeriol-O-malonylhexoside contributed more to the non-enzymic antioxidation.

Abscisic acid functions as a major hormone for regulating plant stress response and adaptation for drought. The ABA signaling is conserved across plants (Chen et al., 2021). However, as demonstrated by the present and other studies (An et al., 2019), the accumulation patterns of ABA and genes involved in this signaling vary according to the varieties and strength of drought stress. It is proposed that mitogen-activated protein kinase *TaMPK3* suppresses ABA response by destabilizing *TaPYL4* receptor in wheat (Liu et al., 2022). In the present study, the expression patterns of *MPK3* and *PYL4* showed opposite trend (Figure 7), providing evidences for such conclusion at transcriptional level. ABA and the genes *MPK3*, *snRK2*, *PYL4*, and *ABF* in *Mu* slightly responded to moderate drought stress and strongly to severe drought stress, while those in CK responded differently. We believe that proper responses of ABA and genes involved in ABA signaling may bring about drought tolerance, which is owed to increased antioxidant competence and decreased ROS accumulation, as demonstrated by the antioxidase activities and MDA content. That is, genes participating ABA signaling in drought-tolerant cultivars are more prone to show linear regulation thanks to the homeostasis mechanisms, which may separate plants with different drought tolerance (Rossdeutsch et al., 2016).

Stomata closure under drought leads to disruption of the supply of parenchyma cells with carbon dioxide, and consequently impacts the efficiency of photosynthesis by inhibiting carbon assimilation and light reactions (Razi and Muneer, 2021). Besides degradation of photosynthetic pigments, drought negatively affects the whole photosynthetic apparatus (López-Jurado et al., 2016). Concurrent with this, in the present study, a variety of genes participating PSI, PSII, cytochrome b6/f complex, photosynthetic electron transport, F-type ATPase, and light harvesting chlorophyll II protein complex were found to be down-regulated (Supplementary Figure S4). In addition, GO enrichment showed that almost all the top 10 most enriched terms were photosynthesis related (Figure 9B), suggesting that photosynthesis was greatly affected by drought. It is deemed that rehydration would not render great difference between CK and *Mu*, since the strategies for coping with drought seemed to be similar in the two lines. To our surprise, however, the photosynthesis in *Mu*4 recovered more thoroughly than that in CK4, since less DEGs were identified in *Mu* (Figure 8). Besides photosynthesis, ribosomal related biological processes were significantly enriched in *Mu* during rehydration as compared to *Mu*1. Only biosynthesis of secondary metabolites pathway was enriched in CK during recovery. We suggest that the difference may be attributed to the fact that severe drought caused irreversible damage to CK, but not to *Mu*, such that *Mu* could take advantage of the previously decomposed amino acids to recover growth after rehydration. However, this needs to be further validated through detecting growth indicators.

Correlation analysis between the metabolome and transcriptome is an effective method for identifying genes involved in drought response (Li et al., 2019). The correlation results between the metabolome and transcriptome revealed that the biosynthesis of amino acids and its downstream pathways, and biosynthesis of secondary metabolites were the co-regulated pathways during drought stresses and rehydration (Table 1). Further, the network constructed based on the pathways showed that shikimic acid, L-phenylalanine and N-Acetyl-L-glutamic acid were positively correlated with genes encoding FBA, P5CS, and CS. These genes were reported to regulate the tricarboxylic acid cycle or respond to drought stresses (Xie et al., 2019). Therefore, the up- or down-regulation of amino acids directly or indirectly provided substrates or energy for biosynthesis of drought response substances to alleviate the energy deficiency caused by decreased photosynthesis and ROS damage.

**Figure 11:**
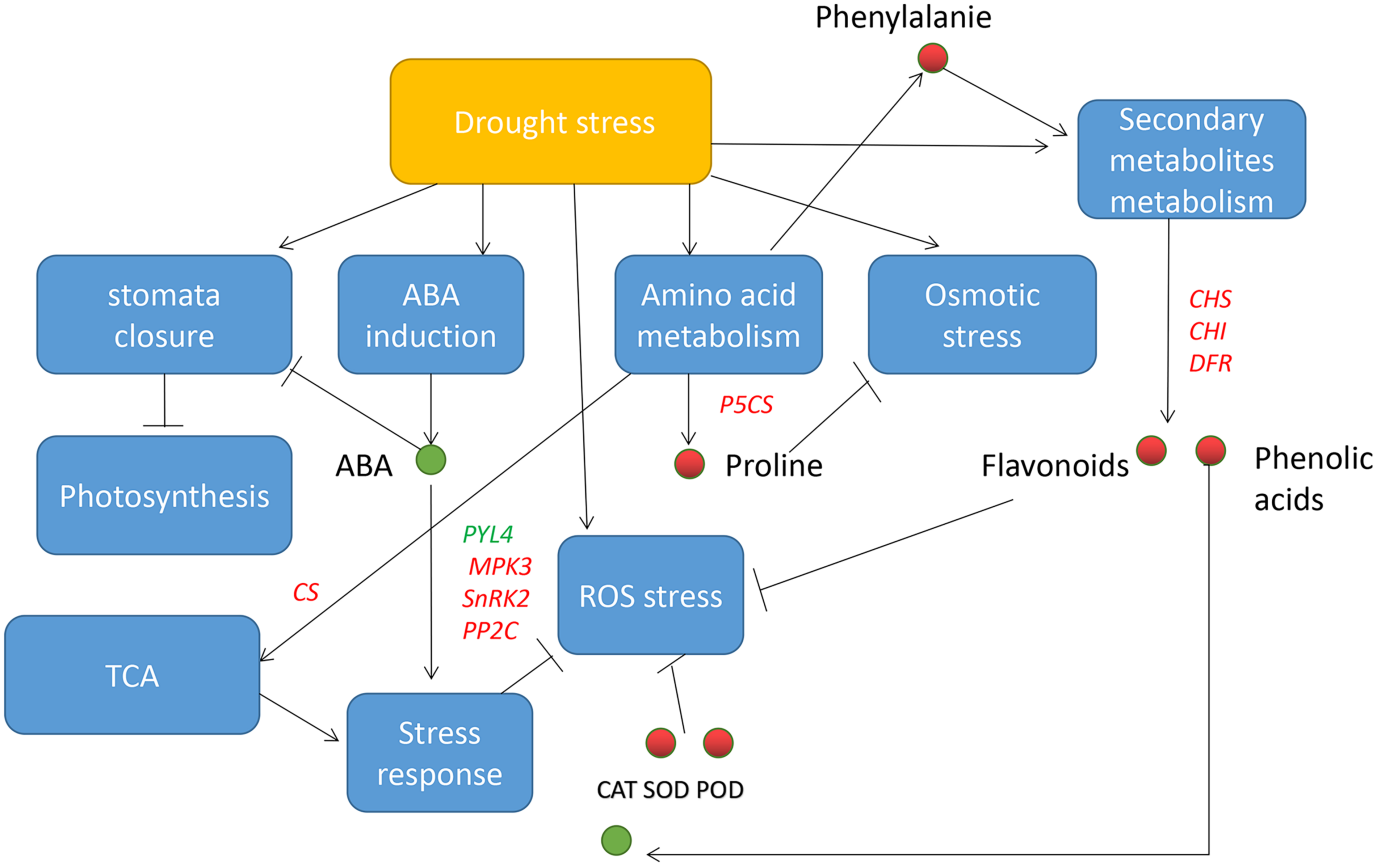
Drought tolerance model based on the present study. Blue rectangle indicates biological process, red/green circle indicates increased/decreased substances, red/green words indicates up-/down-regulated genes, line (T) indicates inhibition, and arrows indicates induction.

Collectively, *Mu* showed greater drought tolerance and recovery capability. We propose that increased amino acids biosynthetic capability and ROS scavenging ability resulted from higher antioxidase activities and increased flavonoids and phenolic acid may be the mechanisms underlying the drought tolerance characteristic of *Mu*. The above mechanisms further change the regulatory profile of drought response genes and metabolites (Figure 11). In addition, recovery from reversible ROS damage and rapid amino acid biosynthesis may contribute to the rapid recovery of *Mu*.

## Data availability statement

The data presented in the study are deposited in the SRA repository, with the accession number PRJNA801431 and PRJNA800168.

## Author contributions

AZ: conceived the project and set the scientific objectives. LL, XC, HL, and JH: contributed to the preparation of equipment and acquisition of data. LL: wrote the manuscript. YL and AZ: reviewed and edited the manuscript. All authors contributed to the article and approved the submitted version.

## Funding

This research was supported by the Natural Science Foundation of Hebei Province (C2020301004), HAAFS Science and Technology Innovation Special Project (2022KJCXZX-LYS-2), Earmarked Fund for Hebei Wheat Innovation Team of Modern Agro-industry Technology Research System (21326318D), and Key R&D project of Hebei Province (20326313D).

## Conflict of interest

The authors declare that the research was conducted in the absence of any commercial or financial relationships that could be construed as a potential conflict of interest.

## Publisher’s note

All claims expressed in this article are solely those of the authors and do not necessarily represent those of their affiliated organizations, or those of the publisher, the editors and the reviewers. Any product that may be evaluated in this article, or claim that may be made by its manufacturer, is not guaranteed or endorsed by the publisher.

## Footnotes

^1^ ftp://ftp.ensemblgenomes.org/pub/plants/release-31/fasta/triticum_aestivum/dna/

^2^ https://www.genome.jp/kegg/

^3^ http://www.geneontology.org/
