# Supplementary material for: Different adaptive patterns of wheat with different drought tolerance under drought stresses and rehydration revealed by integrated metabolomic and transcriptomic analysis
Source: Front Plant Sci. 2022 Oct 13;13:1008624. doi: 10.3389/fpls.2022.1008624 (PMC9608176; doi:10.3389/fpls.2022.1008624)
Supplement: Supplementary file 8 [file Table_8.DOCX]

Supplementary Material


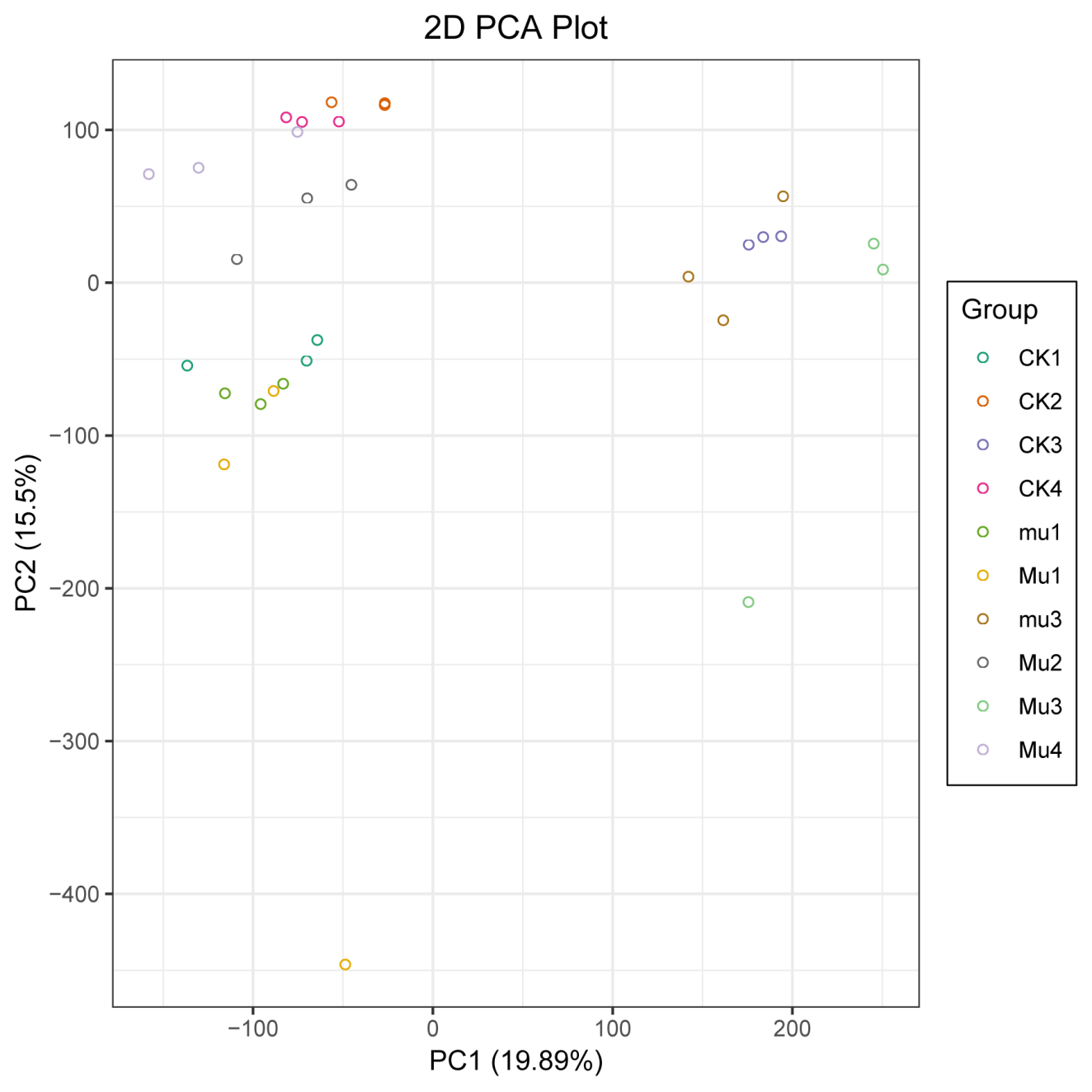


**Supplementary Figure 2.** Soluble sugar content of the three cultivars under drought stresses and after rehydration.
