# Supplementary material for: Different adaptive patterns of wheat with different drought tolerance under drought stresses and rehydration revealed by integrated metabolomic and transcriptomic analysis
Source: Front Plant Sci. 2022 Oct 13;13:1008624. doi: 10.3389/fpls.2022.1008624 (PMC9608176; doi:10.3389/fpls.2022.1008624)
Supplement: Supplementary file 9 [file Table_9.DOCX]

Supplementary Material


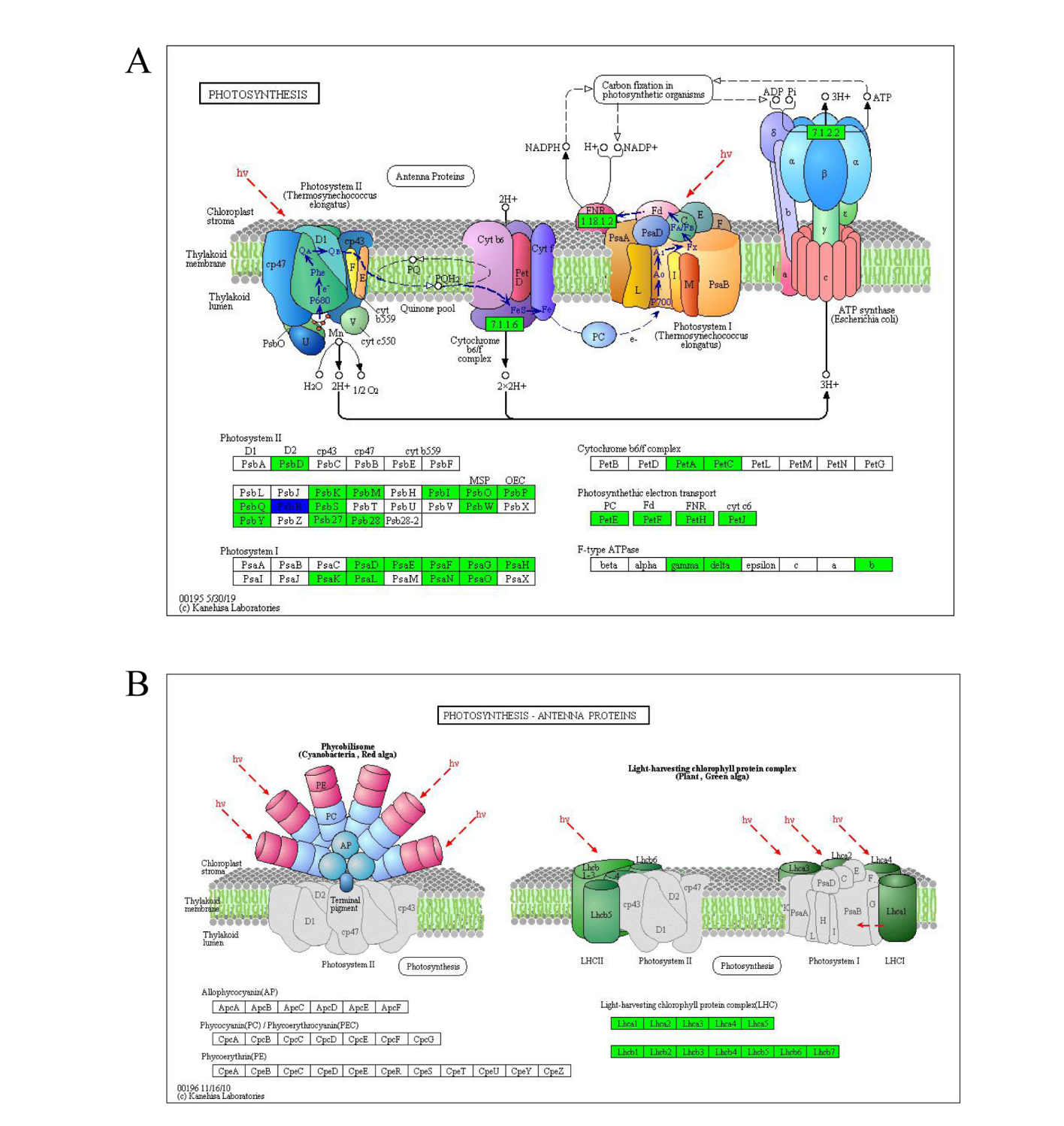


**Supplementary Figure 3.** DEGs involed in photosynthesis in Mu during severe drought. **(A)** DEGs involved in photosynthesis during rehydration. **(B)** DEGs involved in the ‘photosynthesis’ pathway during severe drought in Mu. DEGs involved in the ‘photosynthesis-antenna proteins’ pathway during severe drought Mu
