# Supplementary material for: Different adaptive patterns of wheat with different drought tolerance under drought stresses and rehydration revealed by integrated metabolomic and transcriptomic analysis
Source: Front Plant Sci. 2022 Oct 13;13:1008624. doi: 10.3389/fpls.2022.1008624 (PMC9608176; doi:10.3389/fpls.2022.1008624)
Supplement: Supplementary file 10 [file Table_10.DOCX]

Supplementary Material

| Symbol | Accession number |  | Primer |
| --- | --- | --- | --- |
| bZIP39 | TraesCS1A02G258100 | F | TGAAGGAATGGCAGGGCCTATC |
|  |  | R | GCGGAAATGTCAAACTGAAACACC |
| CCR | TraesCS1D02G198500 | F | CGCTACCTCTGCTTCAACAATGTC |
|  |  | R | GGATCACCTTCAGCTCGTCACTTG |
| P5Cs | TraesCS3A02G363700 | F | CCATCCCGATACGAAACTAATGCC |
|  |  | R | AAAGGAACGGCAGCGAACAG |
| PP2C | TraesCS3D02G249000 | F | TGCTAAGAGAGGTGCTGAGAGC |
|  |  | R | ATGGTTCCTTCACGTCCACCTC |
| MAPK5 | TraesCS4B02G197800 | F | CGCTCCAACCAAGAACTCTCAG |
|  |  | R | TAGCAGGTTGCTCGGTTTCAGG |
| GDSL | TraesCS7B02G013200 | F | AGTGGTTCCAGACCATCACCTC |
|  |  | R | CGAAGATGAAGAGCGAGTTGGC |
| WRKY71 | TraesCS6B02G175100 | F | GCGTCCATTCCATTACAGTAGAGC |
|  |  | R | TGGTGGTGGCTGTGCGGGT |
| MYB44 | TraesCS5B02G157300 | F | ATCGGCATCGCCTGCTGAAG |
|  |  | R | TGAAGCACAGCCCCGGGTAT |
| CPK10 | TraesCS5A02G426500 | F | CAACGCCAACGCCAATGGA |
|  |  | R | GCCTTGCTCACGATGTGGC |
| CAT | TraesCS6B02G056800 | F | CATGTGATGTGGATGGATGATGG |
|  |  | R | CGGTAGGTAATCGACCACATG |
| ACTIN | TraesCS1A02G020500 | F | GGAAAGTGCAGAGAGACACG |
|  |  | R | TACAGTGTCTGGATCGGTGGT |

**Supplementary Table 1.** Primers used for qRT-PCR validation.
